# Comparative proteomics of soluble factors secreted by human breast adipose tissue from tumor and normal breast

**DOI:** 10.18632/oncotarget.25749

**Published:** 2018-07-24

**Authors:** Sabrina Johanna Fletcher, María Belén Hapon, Eduardo A. Callegari, María Luján Crosbie, Natalia Santiso, Anabela Ursino, Alicia Rita Amato, Alberto Gutiérrez, Paula Alejandra Sacca, Rubén Dreszman, Adriana Pérez, Rubén Walter Carón, Juan Carlos Calvo, Virginia Pistone-Creydt

**Affiliations:** ^1^ Laboratorio de Química de Proteoglicanos y Matriz Extracelular, Instituto de Biología y Medicina Experimental (IBYME), Buenos Aires, Argentina; ^2^ Laboratorio de Hormonas y Biología del Cáncer, Instituto de Medicina y Biología Experimental de Cuyo (IMBECU), Centro Científico y Tecnológico Mendoza, Consejo Nacional de Investigaciones Científicas y Técnicas (CONICET), Mendoza, Argentina; ^3^ University of South Dakota, Sanford School of Medicine, Vermillion, South Dakota, USA; ^4^ Sección de Patología Mamaria, Servicio de Ginecología, Complejo Médico Policial “Churruca-Visca”, Buenos Aires, Argentina; ^5^ Clínica de Microcirugía, Buenos Aires, Argentina; ^6^ Departamento de Ecología, Genética y Evolución, Facultad de Ciencias Exactas y Naturales, Universidad de Buenos Aires, Buenos Aires, Argentina; ^7^ Departamento de Química Biológica, Facultad de Ciencias Exactas y Naturales, Universidad de Buenos Aires, Buenos Aires, Argentina; ^8^ Departamento de Fisiología, Universidad Nacional de Cuyo, Facultad de Ciencias Médicas, Mendoza, Argentina; ^9^ Laboratorio de Reproducción y Lactancia, Instituto de Medicina y Biología Experimental de Cuyo (IMBECU), Centro Científico y Tecnológico Mendoza, Consejo Nacional de Investigaciones Científicas y Técnicas (CONICET), Mendoza, Argentina

**Keywords:** human breast cancer, adipose tissue, epithelial-stromal interaction, proteomics analysis, tumor microenvironment

## Abstract

Tumor progression depends on the tumor-stroma interaction. In the breast, adipose tissue is the predominant stromal type. We have previously demonstrated that conditioned media (CMs) from explants of human adipose tissue of tumor breasts (hATT) increase proliferation and migration of breast cancer epithelial cells when compared to human adipose tissue from normal breasts (hATN). In this work, we aim to identify specific proteins and molecular/biological pathways associated with the secretion profile of hATT and hATN explants.

hATT-CMs and hATN-CMs were separated by SDS-PAGE and analyzed by means of two-dimensional nano-liquid chromatography-mass spectrometry. The data was analyzed using ProteoIQ and FunRich software. In addition, 42 cytokines from hATT-CMs and hATN-CMs were assayed by a protein antibody assay. Compared to hATN-CMs, hATT-CMs showed greater protein diversity. We found that hATT-CMs presented a greater amount of proteins related to complement system activity, metabolism and immune system, as well as proteins involved in a variety of biological processes such as signal transduction and cell communication. Specifically, apolipoprotein AI and AII, complement component 3, and vimentin and desmin were significantly increased in hATT-CMs versus hATN-CMs. Moreover, a multivariate discriminant analysis of the cytokines detected by the array showed that IL-6, MCP-2 and GRO cytokines were sufficient and necessary to differentiate hATT-CMs from hATN-CMs. This analysis also showed that the levels of these three cytokines, taken together, correlated with stage and histological grade of the tumor in the hATT-CMs group, and with body mass index in the hATN-CMs group.

## INTRODUCTION

An essential information exchange is established between epithelial tissue and fibroblastic/adipose stroma during normal morphogenesis and functionality of the breast, as well as in cancer development. A deep understanding of this epithelial-stromal interaction could help to unravel new pathways and processes involved in tumor progression. Tumor microenvironment is one of the emerging topics of interest among oncology researchers [1–5]. Interactions of tumor cells with fibroblasts, immune cells and endothelial cells have been subject of extensive studies in different types of cancer, including breast, prostate and melanoma [6–9]. However, adipocytes and adipose tissue have recently started to gain attention as a key component of the tumor stroma [10–13]. Adipose tissue is a bioactive endocrine organ that secretes soluble factors and contributes significantly to the composition of the extracellular matrix (ECM). Adipocytes can influence tumor progression through the secretion of lipids, adipokines, cytokines and hormone conversion mediated by aromatases [14–16]. We have recently shown that conditioned media (CMs) from explants of human adipose tissue from tumor breasts (hATT) increase the proliferation and migration of breast cancer epithelial cell lines, as opposed to CMs from explants of adipose tissue from normal breasts (hATN) [17]. We also demonstrated that adipocytes from the tumor microenvironment show a less differentiated state than adipocytes from a normal microenvironment. This would indicate a loss of normal functions of adipocytes (such as energy storage), and the acquisition of others that might favor tumor growth [18]. Some groups have studied the secretome from tumor breast adipose tissue and interstitial fluid from fresh adipose tissue from cancer patients [19, 20]. However, in both cases, the breast adipose tissue was only from a tumor microenvironment origin. The comparison if this secretome with one from breast adipose tissue of healthy women remains pending. In the present work, we aim to characterize proteins and molecular/biological processes and pathways associated with differences between CMs of human adipose tissue from normal (hATN) and tumor (hATT) breasts. We performed, to our knowledge, the first proteomic analysis which compares breast adipose tissue from healthy women with that of a tumor microenvironment from cancer patients.

The identification of factors that are differentially secreted by normal and tumor breast adipose tissue, and the study of their possible involvement in the regulation of tumor progression, might help develop new strategies to prevent/detect and/or treat breast cancer.

## RESULTS

### hATT-CMs have greater protein diversity than hATN-CMs

**Figure 1:**
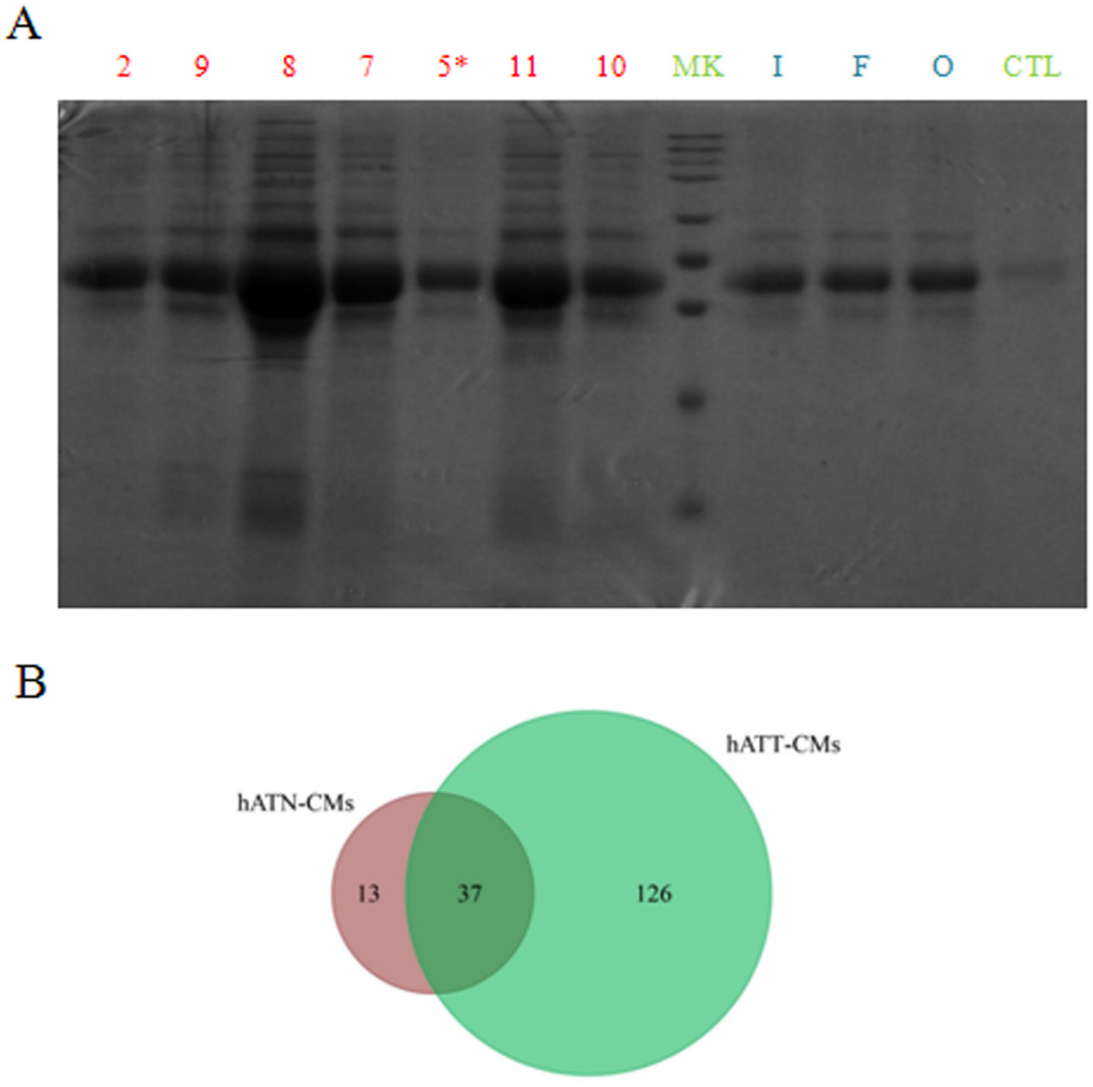
Comparison of protein composition and diversity of hATT-CMs and hATN-CMs **(A)** Proteins present in 100 μl of hATT-CM (n=7) and hATN-CMs (n=3) were separated in a SDS-polyacrylamide gel dyed with Coommassie Brilliant Blue. hATT-CMs: samples 2, 9, 8, 7, 5^*^, 11 and 10 (Sample 5^*^ was excluded from further analysis because it belongs to a male breast cancer patient). hATN-CMs: samples I, F and O. CTL: control-CM. MK: molecular weight markers. **(B)** Aliquots of 30 μg of the same CMs were analyzed by 2D-nanoLC-MS/MS. Venn diagram showing unique and common proteins between hATN-CMs (n=3) and hATT-CMs (n=3).

To begin with the identification and characterization of the different set of proteins present in the CMs, we separated the proteins of hATT-CMs and hATN-CMs in large polyacrylamide gels. In these gels, we observed that hATT-CMs present a greater number of bands compared to hATN-CMs (Figure 1A), indicating a greater protein diversity. Furthermore, aliquots from the same CMs were analyzed by means of two-dimensional nano-liquid chromatography-mass spectrometry (2D-nanoLC-MS/MS) with the aim of identifying specific proteins. The *in silico* analysis of the data was performed with ProteoIQ and FunRich software. Interestingly, the analysis confirmed that hATT-CMs present higher protein diversity than hATN-CMs and also revealed that only 22.7% of the proteins detected in hATT-CMs are common with hATN-CMs (Figure 1B). In addition, the results showed that 97.5% in hATT-CMs and 100% in hATN-CMs of the total proteins identified are normally found in plasma, which confirms that the CMs are mostly composed of secreted or extracellular factors (the full set of proteins detected are listed in Supplementary Table 1 for hATT-CMs and Supplementary Table 2 for hATN-CMs).

### Biological processes and pathways associated with differences between hATT-CMs and hATN-CMs

**Figure 2:**
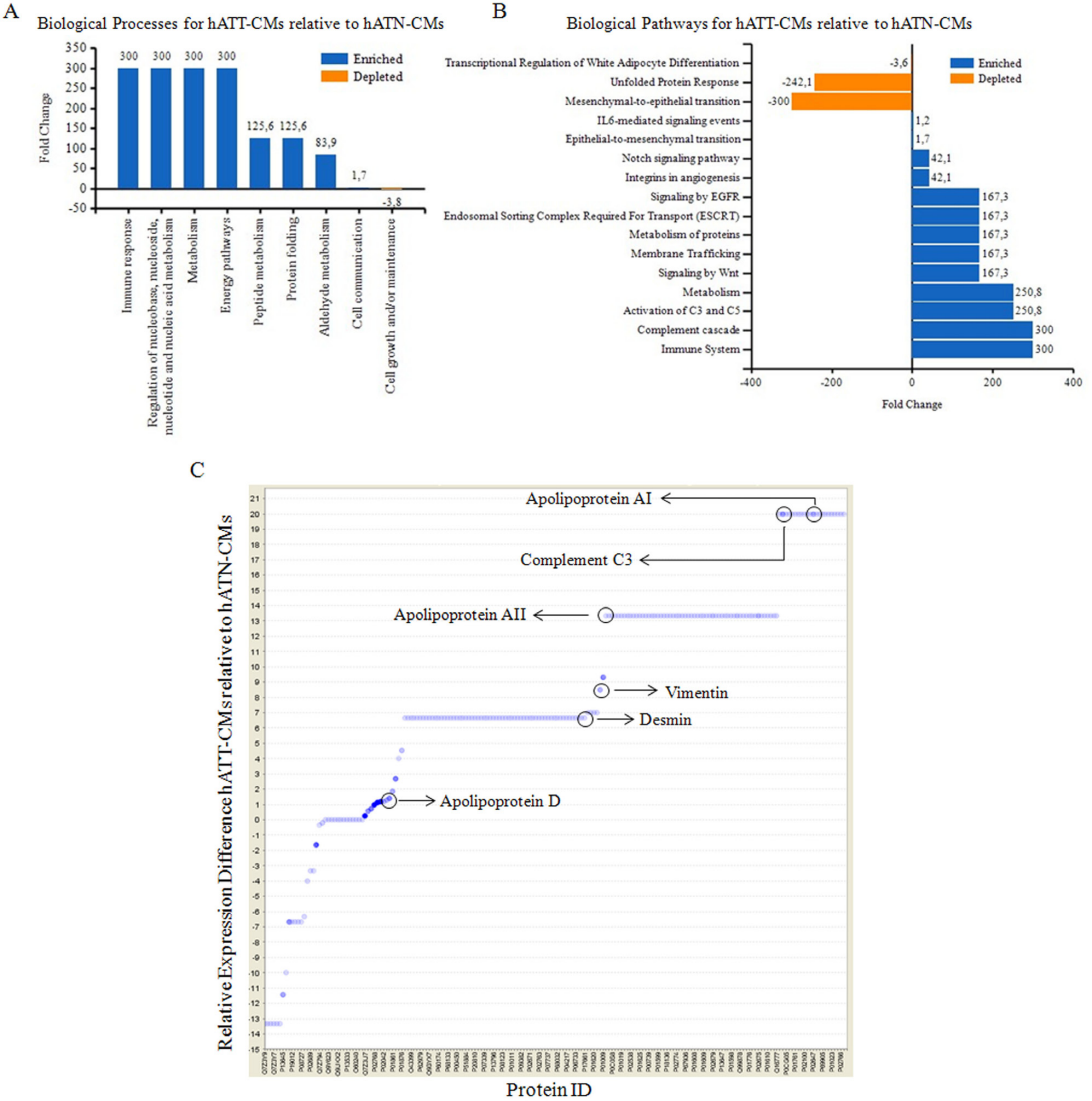
Analysis of biological processes/pathways present in the secretome of hATN and hATT Relative expression difference in specific proteins. *In silico* analysis of data from 2D-nano-LC-MS/MS of hATN-CMs (n=3) and hATT-CMs (n=3). **(A)** Column chart representing fold change in biological processes present in hATT-CMs relative to hATN-CMs. **(B)** Bar chart representing fold change in biological pathways present in hATT-CMs relative to hATN-CMs. **(C)** Proteins in hATT-CMs (n=3) and hATN-CMs (n=3) were identified by 2D-nano-LC-MS/MS and relative changes in the presence of the identified proteins were analyzed. Dot blot represents relative changes of each protein (Protein ID) in hATT-CMs compared to hATN-CMs. Key proteins are marked.

An overall analysis of the processes and pathways represented in the different CMs showed that proteins in both, hATN-CMs and hATT-CMs, were assigned to processes such as cell growth and/or maintenance, metabolism, cell communication, transport and signal transduction (data not show). Furthermore, a comparison between the processes represented in the hATT-CMs versus the hATN-CMs revealed that hATT-CMs expressed more proteins related to immune response, peptide and aldehyde metabolism and cell communication (Figure 2A). In addition, we found that hATT-CMs were enriched in proteins related to complement system activity, metabolism, membrane trafficking and epithelial-to-mesenchymal transition, among others. On the other hand, hATT-CMs had lesser amount of proteins related to mesenchymal-to-epithelial transition and white adipocyte differentiation pathways (Figure 2B).

Furthermore, several different proteins were found overrepresented in hATT-CMs versus hATN-CMs. Among these, a set of apolipoproteins, which are proteins involved in lipid transport and metabolism, were overexpressed in hATT-CMs compared to hATN-CMs (Figure 2C). Particularly, we found overexpressed apolipoprotein AI and AII (20-fold and 13.5-fold change) which are involved in lipid metabolic processes and apolipoprotein D (1.5-fold change), marker for senescent cells. In addition, hATT-CMs were also enriched in complement component 3 (C3) (20-fold change), involved in the activation of the complement system, and in vimentin and desmin (8.5-fold and 6.5 fold change), which are mesenchymal cell proteins related to an invasive breast cancer phenotype.

Therefore, we confirm that the adipose tissue surrounding the tumor would stimulate tumor progression by secreting pro-tumorigenic factors.

### IL-6, GRO and MCP-2 are necessary and sufficient to differentiate hATT-CMs from hATN-CMs

**Figure 3:**
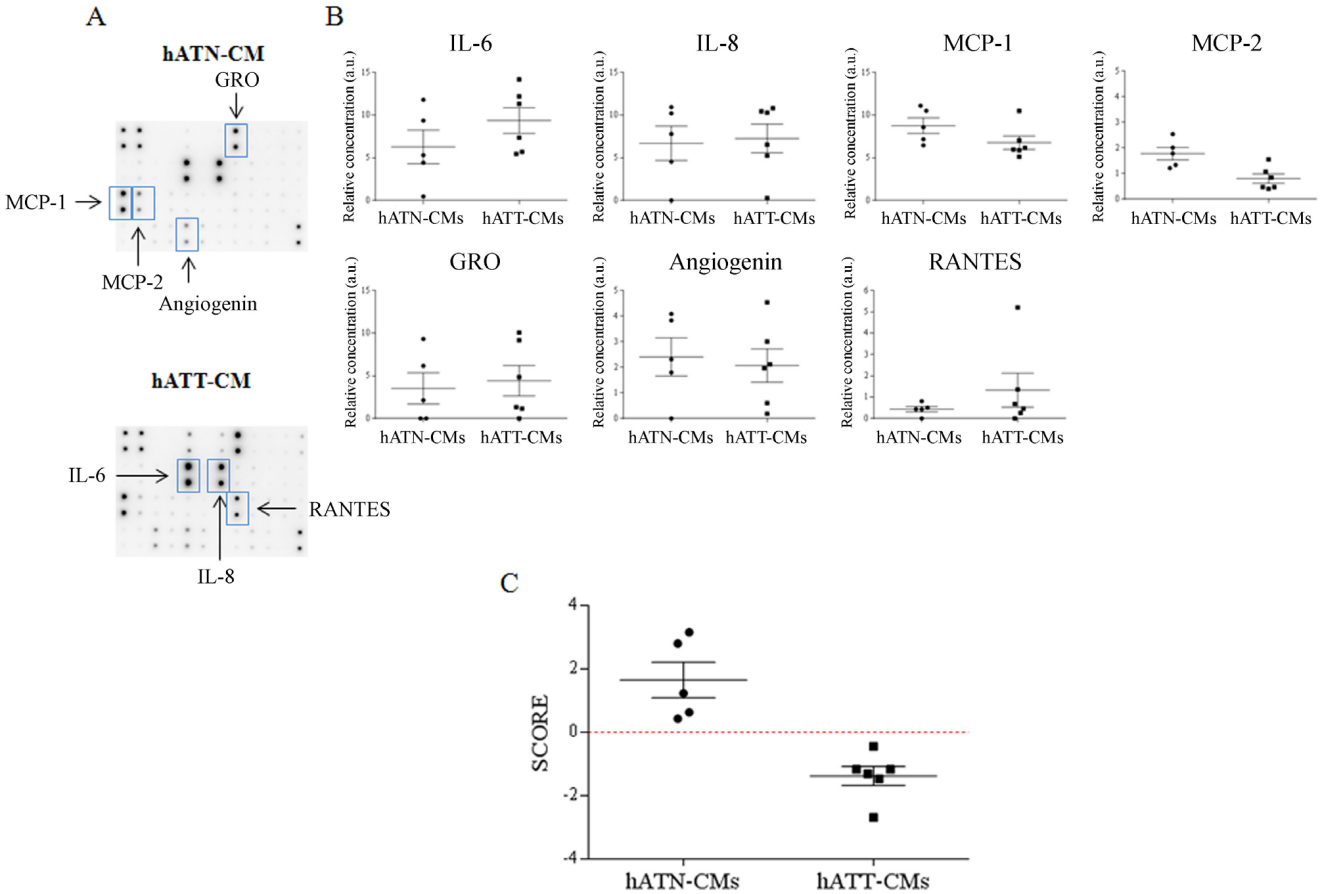
IL-6, MCP-2 and GRO as key cytokines that differentiate the secretome of hATT from hATN hATT-CMs (n=6) and hATN-CMs (n=5) were assessed against a 42 Cytokines Antibody Array. **(A)** Representative images of a membrane incubated with 1mL of hATN-CM or hATT-CM. Rectangles mark relevant cytokines. **(B)** Univariate analysis comparing the levels of IL-6, IL-8, MCP-1, MCP-2, GRO, angiogenin and RANTES from the array between hATN-CMs and hATT-CMs. Scatter plot shows mean ± SEM. a.u.: arbitrary units. Even though no significant difference was found, a tendency was seen in IL-6, MCP-1 and MCP-2. **(C)** A Multivariate Discriminant Analysis of the cytokines from (B)showed that IL-6, MCP-2 and GRO were sufficient and necessary to differentiate hATT-CMs from hATN-CMs. Score: score assigned to each CM from the discriminant analysis. A more positive score represents higher levels of MCP-2 and GRO and lower levels of IL-6.

To complete the proteomics analysis, we used an antibody array to test 6 hATT-CMs and 5 hATN-CMs against 42 specific cytokines. We found that IL-6, IL-8, MCP-1 (CCL2), GRO (alpha, beta and/or gamma) and angiogenin presented the higher levels from the 42 cytokines evaluated both in hATT-CMs and hATN-CMs (Figure 3A and Supplementary Figure 1). MCP-2 (CCL8), and RANTES (CCL5) were also found in at least one of the CMs. The univariate analysis for each of these cytokines showed that IL-6 tended to be increased in hATT-CMs versus hATN-CMs. Interestingly, the opposite was found with MCP-1 and MCP-2, which showed a tendency to be decreased in the hATT-CMs when compared to the hATN-CMs (Figure 3B). We did not find a significant difference between these cytokines, however this could be due to the small number of samples tested and because of the number of variables that are being tested at the same time. Taken this into consideration, we next tested the relevance of these cytokines by evaluating if some of them could be sufficient to differentiate the group of hATT-CMs from hATN-CMs. For this, we performed a multivariate discriminant analysis starting with the 7 cytokines listed above. The multivariate analysis showed that IL6, GRO (alpha, beta and/or gamma) and MCP-2 were necessary and sufficient to differentiate hATT-CMs from hATN-CMs (Figure 3C). Surprisingly, we found that the score assigned to each CM, which comprises the levels of these cytokines taken together, significantly correlated with tumor stage and histological grade (HG) of hATT-CMs (Table 1) and with body mass index (BMI) of hATN-CMs (Table 2) (p<0.05).

**Table 1:** Correlation of hATT-CMs score with tumor stage and histological grade

hATT-CM	Score	Stage	HG
“25”	-2.69	IIb	HG3
“20”	-1.47	IIa	HG3
“22”	-1.31	I	HG2
“30”	-1.17	I	HG2
“19”	-1.17	I	HG2
“27”	-0.44	I	HG1

Score in the canonical axis of each hATT-CM from the discriminant analysis (Figure 3C) correlated with tumor stage and histological grade (HG). Nonparametric Spearman correlation test was performed between score and tumor stage and between score and HG. In both cases, correlation was significant (p<0.05). A more negative score represents a hATT sample more different from the hATN group. Thus, the sample with a score closer to 0 (“27”) is the most similar within the hATT-CM to a hATN-CM and this goes in line with the lower tumor stage and HG of the tumor from that patient.

**Table 2:** Correlation of hATN-CMs score with body mass index

hATN-CM	Score	BMI
“AE”	3.16	21.08
“AD”	2.8	22.31
“AG”	1.23	24.46
“AF”	0.63	25.39
“X”	0.43	29.3

Score in the canonical axis of each hATN-CM from the discriminant analysis (Figure 3C) correlated with body mass index (BMI). Nonparametric Spearman correlation test was performed between score and BMI. Correlation was significant (p<0.05). A more positive score represents a hATN sample more different from the hATT group. As the score approaches to 0, the hATN-CM sample is more similar to the hATT-CMs group and, at the same time, the BMI of that women increase.

## DISCUSSION

Epithelial-stromal interactions mediate the development and progression of breast cancer. Adipocytes are the predominant stromal cell type in breast tissue. We have recently demonstrated that breast adipose tissue from a tumor microenvironment, through the release of soluble factors, can influence the behavior of breast cancer cells [17, 18]. The present work aims to further characterize this bidirectional communication by identifying and comparing the secretome of human adipose tissue explants from normal (hATN) and tumor (hATT) breasts. Therefore, we performed, to our knowledge, the first proteomic analysis comparing human adipose tissue from normal and tumor breasts.

Nowadays, large-scale proteomics techniques allow identifying and comparing the protein composition of extremely complex protein samples. Therefore, in order to analyze and compare secretion profiles from hATT and hATN, we performed a proteomics study of hATT-CMs and hATN-CMs. On the one hand, we found that almost all proteins detected in hATT-CMs and hATN-CMs are intrinsic to plasma/serum. Thus, these proteins can be normally found as extracellular components.

In addition, we found that hATT-CMs present an increased protein diversity compared to hATN-CMs. This result was obtained by protein separation in polyacrylamide gels and 2D-nanoLC-MS/MS analysis (Figure 1A). Furthermore, hATN-CMs samples showed a very similar secretion profile between them, while hATT-CMs samples showed higher diversity. This result agrees with what we found when employing the CMs in different functional assays, as shown in our previous work [18].

Furthermore, the comparison of the proteomic profiles between hATT-CMs and hATN-CMs showed that some biological processes and pathways are increased in hATT-CMs compared to hATN-CMs (such as immune response and several metabolic pathways, Figure 2A-2B). C3 from the complement system was one of the proteins detected involved in the immune response [21], and its activation is associated with an increase in local inflammation. Several works have found a relation between local inflammation and the presence of a tumor [22, 23]. In fact, inflammation of tumor microenvironment has been shown to induce a “reactive” tumor stroma, were events like proliferation, invasion and metastasis are stimulated [24].

We also found that hATT-CMs present increased levels of several proteins involved in lipid metabolism and transport such as apolipoproteins AI and AII (Figure 2C). All apolipoproteins can transport lipids, including fatty acids (FAs) [25]. In fact, there is evidence that in this way adipocytes may release triglycerides and FAs, that are then taken as energy source by nearby tumor cells [26, 27]. Furthermore, Wang *et al.*, using an *in vitro* co-culture system, recently demonstrated that a metabolic symbiosis occurs between adipocytes and breast cancer tumor cells [12]. Our present results are therefore in accordance with previous works, suggesting that adipose tissue from tumor microenvironment could be “feeding” tumor cells through the release of lipids.

In addition, the comparative analysis of the data obtained from 2D-nanoLC-MS/MS showed increased levels of vimentin and desmin in hATT-CMs compared to hATN-CMs (Figure 2C). These two proteins are part of the intermediate filaments of the cytoskeleton and are also markers for mesenchymal cells [28]. Vimentin overexpression has been found in several cancer cells, including breast cancer, and this overexpression has been associated to an increase in cell invasion and tumor growth [29]. Although the role of vimentin as an extracellular component is not yet clear, it has been found to be a component of adipose tissue secretome in several works [20, 30]. Furthermore, some groups have found that vimentin can be secreted [31, 32]. On the other hand, desmin has been found to be present in human plasma [33], though no specific cell type has been reported to be responsible for its secretion. However, increased levels of desmin in tumor stroma have been related to advanced stages of colorectal cancer [34]. Nevertheless, there could be an alternative explanation for the presence of classic cytoskeleton proteins in the CMs. We hypothesize that hATT-CMs could have increased levels of exosomes, which are cell-derived extracellular vesicles that play an important role in cell-cell communication [35]. They can transfer genetic material, proteins and lipids from one cell to another [36]. In this regard, cytoskeleton proteins are often found in exosomes. Furthermore, in our proteomics analysis we found that hATT-CMs present a number of histones (Supplementary Table 1), which are usually found in an extracellular localization as components of exosomes [37, 38]. Though more evidence is necessary to validate this hypothesis, our results could be indicating that a specific type of communication occurs between adipose tissue from tumor microenvironment and tumor cells.

Therefore, the comparative analysis of the secretion profiles of hATT and hATN, by means of 2D-nanoLC-MS/MS, indicated that the adipose tissue from tumor microenvironments presented a “reactive” state, and that this adipose tissue secretes increased levels of proteins involved in immunological, metabolic and angiogenic processes, resulting in the promotion of tumor progression.

To further extend the proteomics characterization of the CMs and to identify those proteins that are relevant as components of a tumor or normal breast microenvironment, we used an array against 42 human cytokines (Figure 3). On the one hand, the similarity between the expression profiles (IL6, IL8, MCP-1, GRO and angiogenin) of hATT-CMs and hATN-CMs denotes the likeness in the origin of the obtained adipose tissues. If the cell composition of hATT and hATN had been significantly different, we would have expected greater differences in the cytokine profiles obtained. When we compared differences in the levels of these cytokines, and others less expressed, we did not find a significant difference between the hATN-CMs and hATT-CMs. As stated before, the lack of significant difference could be due to the fact that too many variables (cytokines) are being tested at the same time, and/or because the number of samples of each group is too small. However, we did find a tendency in some cases. In particular, IL-6 seemed to be increased in hATT-CMs compared to hATN-CMs. Several works have described that stromal IL-6 secreted by adipose cells can increase cell migration, invasion and tumor growth [39, 40, 41]. Dirat *et al.* found that cancer-associated adipocytes (CAAs) secrete IL-6 *in vitro* and, using human tissue samples, they showed that this interleukin is increased in tumor stroma, specifically in CAAs [42].

Surprisingly, we found a decrease of MCP-1 and MCP-2 in hATT-CMs compared to hATN-CMs. These two cytokines are involved in macrophage recruitment, and have been previously found to be increased in breast tumors [43, 44]. Furthermore, it has been described that MCP-1 stromal levels correlate with the number of tumor-associated macrophages (TAMs), in a xenographic model of breast cancer [45]. Nevertheless, in the above mentioned work the cellular origin of MCP-1 was not clear, since the authors only distinguished tumor cells from stromal cells, and did not identify which stromal cell type was releasing MCP-1. Therefore, it is possible that our results indicate that adipose tissue is not involved in the release of these cytokines into the tumor stroma.

Since most of the cytokines evaluated in this array can have redundant, synergistic and/or opposite effects, we then evaluated if the levels of some of these proteins taken together could be relevant as differential components of hATT-CMs and hATN-CMs. To test this idea, we performed a multivariate discriminant analysis considering the 7 cytokines detected. The results from the discriminant analysis allowed us to identify IL6, GRO and MCP-2 as sufficient and necessary to differentiate hATT-CMs from hATN-CMs (Figure 3C). In addition, we found that in the case of the hATT-CMs the levels of these three cytokines taken together, represented by the score, correlated with stage and histological grade of the tumor (Table 1). This was a surprising result since what we are comparing are the levels of these cytokines secreted by the adipose tissue from the tumor microenvironment with the stage and differentiation state of the tumor. Moreover, in the hATN-CMs group we found that the score correlated with the BMI of the women (Table 2). This means that the sample from the woman with the higher BMI is the one, among the hATN-CMs group, more similar to a hATT-CM at least in regards to the secretion of the three cytokines. The implication of this result is that the human breast adipose tissue from a healthy overweight woman seems to be behaving more similarly to the human breast adipose tissue from a tumor microenvironment.

In our experimental model we employed whole tissue explants and did not isolate specific cell types. Therefore, although our explants came from breast adipose tissue, there are other cell types present apart from adipose cells (e.g. macrophages, fibroblasts, endothelial cells and immune cells). Nevertheless, adipose cells (preadipocytes, adipocytes and CAAs) are by far the predominant cell type in the explants. This was confirmed analyzing the histology of the explants [18]. In addition, the secretion profiles found for hATT and hATN agreed with secretomes from adipose tissue and adipocytes described by other groups [20, 30].

Our results raise some intriguing questions about the adipose tumor microenvironment. For instance, we wonder if there could be a potential prognostic value of the cytokines IL-6, GRO and MCP-2 or if they could be used as additional markers for breast tumor stage. Of course, further and extensive analyses are required to confirm these results, starting by increasing the number of samples being tested, and also quantifying the actual concentration of the cytokines by ELISA.

The identification of these factors, both in normal and tumor adipose tissue, and the study of their possible involvement in the regulation of tumor progression, might help indentifying new targets for antineoplastic therapies.

## MATERIALS AND METHODS

### Reagents

Reagents were purchased from Sigma Chemical Co (St. Louis, MO, USA), tissue culture flasks and dishes were from Falcon Orange Scientific (Graignette Business Park, Belgium) and NEST Biotechnology (Wuxi, China), culture media and supplements were from Gibco BRL (Carlsbad, CA, USA).

### Sample collection and handling

We collected samples of human breast adipose tissue from both tumor (hATT, n=13) and normal breasts (hATN, n=8). hATT tissue samples were obtained from estrogen and/or progesterone receptor positive, infiltrating ductal carcinomas. None of the patients had received chemotherapy or radiotherapy treatment. The surgeon removed fragments of adipose tissue located approximately 2 cm from the tumor. hATN tissue samples were obtained from plastic surgeries performed for aesthetic reasons (breast reduction). All samples were processed within 2 h under a sterile laminar flow hood. At arrival, tissues were transferred to a Petri dish. If the explants had high and visible amounts of connective tissue and vessels, they were cut-off from the tissue explants. Then, tissues were extensively washed 3 times with ice PBS supplemented with gentamicin (50 μg/ml). The samples were weighed and placed in a culture flask with M199 culture medium (Invitrogen™) (1g tissue/10 mL M199) supplemented with gentamicin (50 μg/mL) and incubated for 1 h at 37°C in 5% CO_2_. After this time, medium was removed and replaced with fresh medium and put back in the incubator. After 24 h CM was collected, centrifuged, filtered, aliquoted into 1 mL fractions and immediately stored at -20°C, until used. M199 medium was used as control-CM. All patients gave their written consent. Samples (tumor and normal) were collected following the approval of the Ethics Committee of both the IBYME and the Complejo Médico Policial “Churruca-Visca”.

### Polyacrylamide gel electrophoresis

100 μL of hATT-CMs (n=7) and hATN-CMs (n=3) were loaded onto a 10% SDS-PAGE gel, and proteins were separated by electrophoresis, at a constant voltage of 100 V. After the run, the gel was fixed with a 50% v/v ethanol, 2% v/v phosphoric acid solution, overnight at 4°C. Then, gel was washed 3 times and proteins were dyed with Coomassie Blue G250 solution (Sigma Chemical Co, St. Louis, MO, USA). Control-CM was used as control and a lane with molecular weight markers was included.

### Protein identification by mass spectrometry analysis

Lyophilized aliquots (30 μg) of hATT- (n=6) and hATN-CMs (n=3) were resuspended in low salt buffer (10 mM HEPES pH7.9, 10 mM KCl, 1.5 mM MgCl_2_) for 15 min on ice and then homogenized using a motorized pestle. The lysate was centrifuged at 4°C at 800xg. The proteins obtained were reduced with 50 mM DTT (Sigma Aldrich, Saint Louis, MO) at 65°C for 5 min, alkylated with 100 mM iodoacetamide (Sigma Aldrich), and digested using sequencing grade trypsin (Promega, Madison, WI) overnight at 37°C. The digestion was stopped by the addition of 0.5% acetic acid, frozen in dry ice, and concentrated using a Savant SpeedVac centrifuge (Thermo Scientific, Hudson, NH). The tryptic-digested peptides were dissolved in 100 mM ammonium formate pH10 and separated through 2D-nanoLC with dilution using a 2D-nanoAcquity UPLC (Waters Corporation, Milford, MA). The first dimension was performed in XBridge BEH130 C18, 5μm, 300μm x 50 mm NanoEase Column (Waters Corporation, Milford, MA) using as solvent A1 20 mM ammonium formate pH10 and B1, 100% acetonitrile (Optima LC/MS, Fisher Scientific, Pittsburgh, PA) LC-MS grade. The flow at 1st dimension was 2uL/min, and 11 different step gradients (dilution method) were performed for 20 min each. The second dimension included trapping and desalting online through 180μm x 20 mm, 5μm symmetry C18 nanoAcquity UPLC trap column (Waters) at a flow 20 uL/min, 99% A2 (water, 0.1% formic Acid), and 1% B2 (100% acetonitrile, 0.1% formic acid) for 20 min. After the peptides were desalted and concentrated, they were separated online in the second dimension through BEH130 C18 1.7 μm, 100 μm x 100 mm nanoAcquity UPLC column. The standard solvent gradient used was: 0-2 min, 3% B2 isocratic; 2-40 min, 3-85% B2 linear, at a flow rate of 400 nL/min for 60 min. The eluted ions were analyzed by one full precursor MS scan (400to1500 m/z) followed by four MS/MS scans of the most abundant ions detected in the precursor MS scan while operating under dynamic exclusion or direct data acquisition system (DDAS). Spectra obtained in the positive ion mode with nano ESI-QTof Synapt G1 mass spectrometer (Waters) were deconvoluted and analyzed using the MassLynx software 4.1 (Waters). A peak list (PKL format) was generated to identify 1 or multiple charged precursor ions from the mass spectrometry data file. The instrument was calibrated in MS/MS mode using 100 fmol of (Glu1)-Fibrinopeptide B human (Sigma) with a RMS residual of 3.857 e^4^ or 6.9413 e^-1^ ppm or 7.722 e^0^ ppm. Parent mass (MS) and fragment mass (MS/MS) peak ranges were 400-1500 Da and 65-1500 Da respectively. Mascot server v2.5.0 and Mascot Daemon Toolbox v2.5.1 (www.matrix-science.com, UK) in MS/MS ion search mode (local licenses) were applied to conduct peptide matches (peptide masses and sequence tags) and protein searches against NCBInr v20150531 (67337701 sequences, 24122812982 residues) taxonomy filter (Homo sapiens) (311623 sequences) and IPI Human v3.80 (86719 sequences, 34928216 residues). The following parameters were set for the search: carbamidomethyl (C) on cysteine was set as fixed; variable modifications included asparagine and glutamine deamidation and methionine oxidation. One missed cleavage was allowed; monoisotopic masses were counted; the precursor peptide mass tolerance was set at 50 ppm; fragment mass tolerance was 0.3 Da, and the ion score or expected cut-off was set at 5. The MS/MS spectra were searched with MASCOT using a 95% confidence interval (C.I. %) threshold (p < 0.05). A minimum score of 46 was used for peptide identification. The protein redundancy that appeared at the database under different gi (protein accession number) was limited to human with the first priority assigned to that species. Data was analyzed using ProteoIQ (Premier Biosoft) and FunRich version 3 software [46]. These software allows to visualize GO and pathways in the generated data.

### Cytokine antibody array

To detect and compare the level of 42 specific cytokines we used a Human Cytokine Antibody Array (Abcam #133997). Experiments were performed following the manufacturer´s protocol. 1 mL of each hATT-CMs (n=6), hATN-CMs (n=3) or control-CM was used to incubated individual membranes. Spots were visualized by means of chemiluminescence and quantified using ImageJ software available at the NIH site [47]. Dots on each membrane were relativized to positive control spots on the same membrane and normalized to control-CM membrane.

### Statistical analysis

For univariate analysis of cytokines from the array, the statistical significance was evaluated by Student´s *t*-test with Bonferroni correction. Results are presented as mean ± SEM. Results were considered significant at p < 0.05, using GraphPad Prism® version 5.01 software (GraphPad Software Inc., CA). Multivariate analysis and nonparametric Spearman correlation test were performed with InfoStat version 2014 [48].

## SUPPLEMENTARY MATERIALS FIGURE AND TABLES







## Abbreviations

2D-nanoLC-MS/MS: two-dimensional nano-liquid chromatography-mass spectrometry
BMI: body mass index
CAAs: cancer-assocciated adipocytes
CMs: conditioned media
ECM: extracellular matrix
EMT: epithelial-mesenchymal transition
FAs: fatty acid
FAK: focal adhesion kinase
FFAs: free fatty acids
hATN: human adipose tissue from normal breast
hATT: human adipose tissue from tumor breast
HG: histological grade
TAMs: tumor-associated macrophages
