# Supplementary material for: Comparative proteomics of soluble factors secreted by human breast adipose tissue from tumor and normal breast
Source: Oncotarget. 2018 Jul 24;9(57):31007–17. doi: 10.18632/oncotarget.25749 (PMC6089553; doi:10.18632/oncotarget.25749)
Supplement: Supplementary file 1 [file oncotarget-09-31007-s001.pdf]

Comparative proteomics of soluble factors secreted by human breast adipose tissue from tumor and normal breast

SUPPLEMENTARY MATERIALS

Supplementary Table 1: Proteins detected for 3 hATT-CMs

See Supplementary Table 1 in Supplementary Files

Supplementary Table 2: Proteins detected for 3 hATN-CMs

See Supplementary Table 2 in Supplementary Files

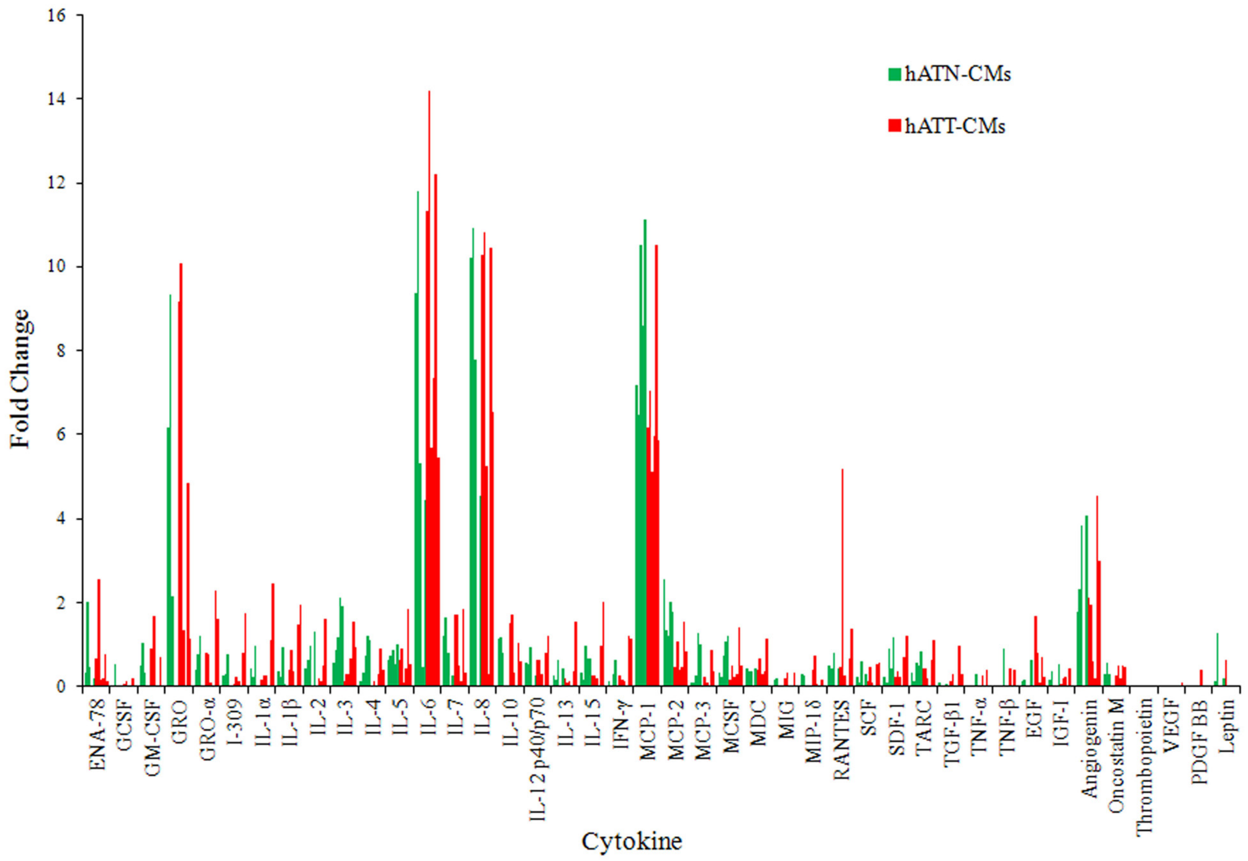

Supplementary Figure 1: Array of 42 cytokines. Dots from the array were measured by densitometry and relativized to control membrane. Bar chart shows relative fold change for each cytokines of hATN-CMs (n=5) and hATT- CMs (n=6).
